# Associated congenital malformations, syndromes, and medical conditions in patients with orofacial clefts: a 10-year hospital-based study in Thailand

**DOI:** 10.3389/froh.2025.1722054

**Published:** 2025-12-18

**Authors:** Krispijyakorn Sangharn, Poonsak Pisek, Araya Pisek, Buddhathida Wangsrimongkol, Waranuch Pitiphat, Agnès Bloch-Zupan, Khunton Wichajarn, Supawich Morkmued

**Affiliations:** 1 Division of Orthodontics, Department of Preventive Dentistry, Khon Kaen University, Khon Kaen, Thailand; 2 School of Pediatric Oral Health, Institute of Dentistry, Suranaree University of Technology, Nakhon Ratchasima, Thailand; 3 International College of Dentistry, Walailak University, Bangkok, Thailand; 4 Division of Dental Public Health, Department of Preventive Dentistry, Khon Kaen University, Khon Kaen, Thailand; 5 Université de Strasbourg, Faculté de Chirurgie Dentaire, Strasbourg, France; 6 Hôpitaux Universitaires de Strasbourg (HUS), Pôle de Médecine et Chirurgie Bucco-dentaires, Centre de Référence des Maladies Rares Orales et Dentaires, CRMR-O-Rares, Filière Santé Maladies Rares TETE COU & European Reference Network ERN CRANIO, Strasbourg, France; 7 Université de Strasbourg, CNRS- UMR7104, INSERM U1258 Institut de Génétique et de Biologie Moléculaire et Cellulaire (IGBMC), Illkirch, France; 8 Université de Strasbourg, Institut d’études Avancées (USIAS), Strasbourg, France; 9 Division of Medical Genetics, Department of Pediatrics, Faculty of Medicine, Khon Kaen University, Khon Kaen, Thailand; 10 Division of Pediatric Dentistry, Department of Preventive Dentistry, Khon Kaen University, Khon Kaen, Thailand

**Keywords:** cleft lip and palate, congenital malformations, medical condition, orofacial clefts, syndromes

## Abstract

**Introduction:**

Orofacial clefts (OFCs) require complex care, which is further complicated by associated congenital anomalies and medical conditions. However, the specific patterns of these associated conditions across different OFC subtypes are not well-characterized in the Thai population. This study aimed to determine the prevalence of OFC subtypes and to analyze the distribution of associated congenital malformations, syndromes, and medical conditions specific to each cleft type.

**Materials and methods:**

We conducted a retrospective analysis of 1,187 patients (0–3 years) treated at Tawanchai Cleft Center, Thailand, between 2011 and 2020. Cases were identified using ICD-10 codes and verified through medical records. Data were analyzed to determine OFC subtype prevalence and characterize the distribution of associated congenital malformations, syndromes, and medical conditions.

**Results:**

Cleft lip and palate (CLP) was the most common subtype (49.2%), followed by isolated cleft palate (CP, 28.1%) and isolated cleft lip (CL, 22.7%). A significant portion of patients (45.4%) presented with at least one associated condition. Respiratory system malformations were most prevalent (35.3%), followed by circulatory (12.2%) and musculoskeletal system anomalies (11.1%). The prevalence of associated malformations was highest in the CP group, which was strongly associated with Pierre Robin Sequence (8.2%). Among the 7% of syndromic cases, 22q11.2 deletion syndrome was the most frequent diagnosis (9.6% of syndromic cases). Medically, otitis media (51.7%) and anemia (17.3%) were significant comorbidities across all groups.

**Conclusion:**

Our findings demonstrate that the profile of associated anomalies differs significantly across OFC subtypes. This underscores the necessity for subtype-specific screening protocols and highlights the high burden of comorbidity in this population, directing a multidisciplinary approach for effective management.

## 1 Introduction

Orofacial clefts (OFCs) represent the most common craniofacial congenital malformations, accounting for approximately 15% of all congenital malformations (1). OFCs encompass a wide range of manifestations and can be classified based on the affected structures as cleft lip (CL), cleft palate (CP), and cleft lip and palate (CLP). Animal models and epidemiologic studies have consistently shown that CLP differs causally and pathogenetically from CP (2). Embryologically, CLP results from the failure of the three facial prominences (lateral nasal, medial nasal, and maxillary prominences) to fuse during the fourth to seventh weeks of gestation. Meanwhile, during the ninth to tenth weeks of gestation, failure of the palatal shelves to fuse results in CP (3).

The etiology of OFCs is complex, involving both genetic and environmental factors, with significant regional and national variations (4). The prevalence of OFCs is higher among Asians (5–8). In Thailand, the most recent population-weighted pooled prevalence of OFCs was reported at 2.14 per 1,000 live births, which is higher than in other regions (9). Among OFC cases, non-syndromic clefts account for 93% of all CL/P cases, while syndromic clefts comprise the remaining 7% (10). Although OFCs typically present as isolated cases, they may occur in conjunction with other congenital malformations, which may be part of either recognized or unrecognized syndromes. Studies have reported that 4.3% to 63.4% of OFC cases are associated with other congenital malformations but that overall the joint symptoms cannot be attributed to a specific syndrome (1, 11, 12).

Medical conditions associated with OFCs pose significant clinical challenges requiring thorough assessment and appropriate therapeutic intervention. For instance, anemia is commonly reported in patients with OFCs, with a prevalence of 29.1% in Thailand, considerably higher than the global prevalence (13). Among children with OFCs in India, anemia affects 74.0% of patients, with iron deficiency in 91.6%, vitamin B12 deficiency in 35.8%, and folate deficiency in 23.5%. Additionally, moderate to severe malnutrition occurs in 53.3% (14). In Burkina Faso, children with OFCs showed anemia, infection, and malnutrition in 39.4%, 9.2%, and 7.5% of cases, respectively (1). Failure to identify and adequately manage these associated medical conditions may compromise the safety and efficacy of primary surgical interventions, specifically cheiloplasty and palatoplasty, by increasing the risk of anesthetic and surgical complications in patients with OFCs (1).

Despite the high incidence of OFCs in Thailand, a significant knowledge gap persists regarding the nationwide epidemiological profile of associated congenital malformations, syndromes, and medical conditions. This absence of comprehensive, large-scale data presents a substantial challenge to the development of evidence-based clinical management protocols. Tawanchai Cleft Center, as the primary referral and treatment hub for Northeastern Thailand, serves a large and ethnically distinct population, making its patient database uniquely representative of this major geographical region. Therefore, data from this center are crucial for addressing the current void in regional and national-level understanding.

Accordingly, this study aimed to leverage a decade of systematically collected data from our center to achieve two primary objectives: (1) to determine the prevalence of each OFC subtype from 2011 to 2020; and (2) to comprehensively characterize the distribution of associated congenital anomalies, genetic syndromes, and medical conditions across these distinct OFC subtypes. By fulfilling these objectives, this study provides a foundational evidence base intended to enhance early diagnosis through targeted screening, inform evidence-based multidisciplinary management, and contribute to the development of updated national guidelines for OFC care in the future.

## 2 Materials and methods

This retrospective hospital-based study was conducted at the Tawanchai Cleft Center, a major tertiary cleft center in Thailand. The center functions as a regional referral hub for patients with cleft lip and/or palate (CL/P) and other craniofacial deformities, providing comprehensive care across the Northeastern region of the country. The primary characteristic of this retrospective design was the reliance on pre-existing clinical data derived exclusively from patient medical records (OPD/IPD charts and discharge summaries), ensuring the capture of real-world clinical documentation. To confirm accurate case identification and data verification, the study relied on the center's multidisciplinary team's expertise. This team comprised specialist doctors, dentists, nurses, and, importantly, a geneticist (KW) who assisted in the precise classification of cases, including those involving complex syndromes and associated congenital anomalies. The study population comprised patients with orofacial clefts (OFCs) aged 0–3 years who were either delivered at or referred to Tawanchai Cleft Center between January 2011 and December 2020.

**Figure 1:**
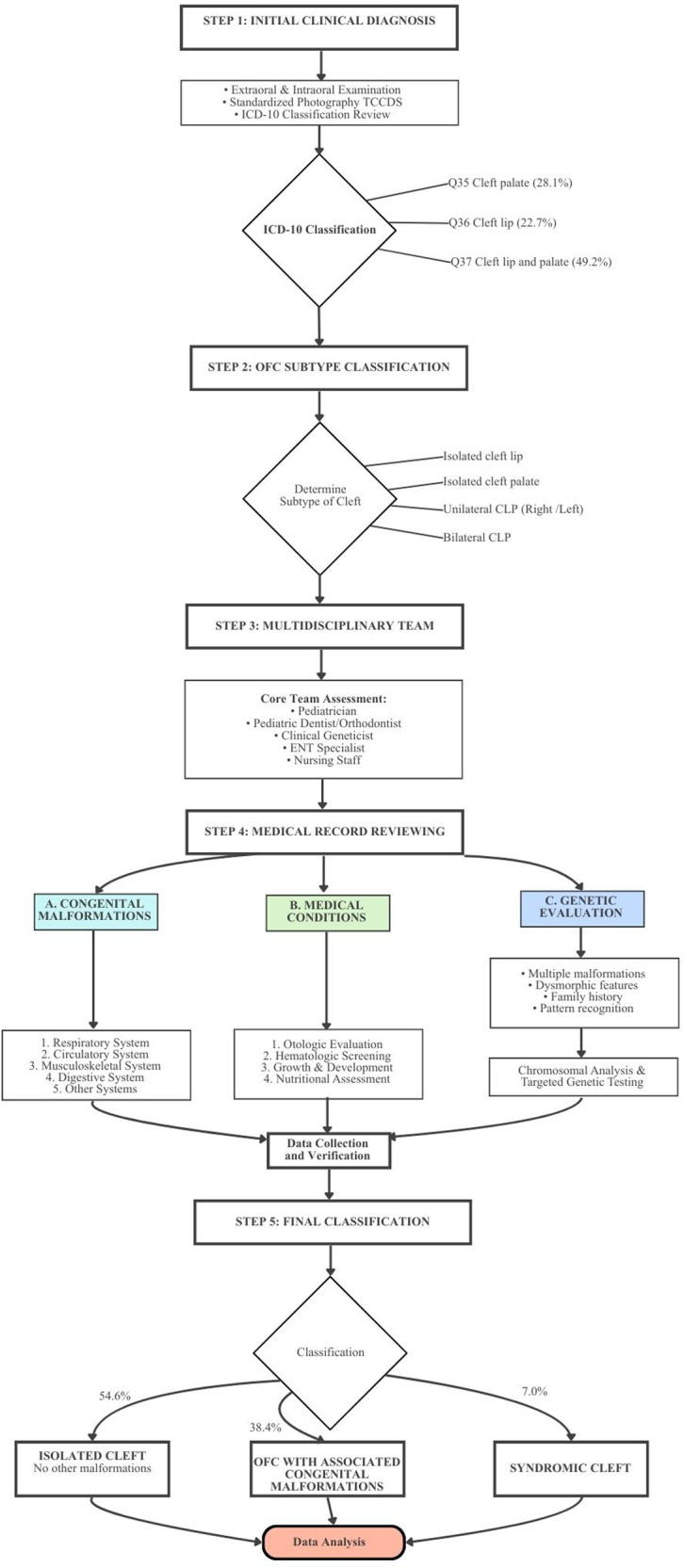
Diagnostic workflow in this study.

Data collection from December 21, 2021, to May 18, 2024, employed a passive case ascertainment approach through the comprehensive review of medical records from the Srinagarind Hospital, as shown in Figure 1. Cases with live births were identified using ICD-10 codes for cleft palate (CP; Q35), cleft lip (CL; Q36), and cleft lip and palate (CLP; Q37). A definitive diagnosis of OFCs was provided by pediatricians, and this diagnostic confirmation served as the foundation for inclusion in the study cohort. Cleft types were subsequently corroborated using standardized intraoral and extraoral photographs archived within the Tawanchai Cleft and Craniofacial Database System (TCCDS). Cases involving syndromes underwent genetic investigation for molecular diagnosis. Both electronic and paper medical records were meticulously reviewed to identify OFC cases with associated congenital malformations, syndromes, and related medical conditions. To ensure consistency and manage the high number of potential variables identified within the retrospective records, careful screening was performed. All extracted data related to congenital malformations and syndromes were cross-referenced with established international classification systems where applicable. This systematic approach minimized ambiguity and ensured that only verifiable, documented diagnoses were retained for final analysis, thus serving to mitigate the risk posed by potential vagueness in the source data.

Case verification involved a thorough review of electronic and paper medical records to confirm birth defect coding, ensure data completeness, and eliminate duplicate records. The inclusion and exclusion criteria were defined as follows:

### 2.1 Inclusion criteria

To be included in the final study cohort, patients had to meet all of the following criteria:

- Primary Diagnosis: A definitive diagnosis of an OFC affecting the lip, alveolus, and/or palate, coded according to the ICD-10 codes Q35-Q37. This diagnosis must have been confirmed by a senior pediatrician or plastic surgeon on the multidisciplinary team.
- Age at Diagnosis: Initial diagnosis or first presentation to our center must have occurred between birth and 3 years of age.
- Data Availability: The patient's medical record must contain, at a minimum:
  ○ Detailed demographic data (date of birth, sex).
  ○ A clear description of the cleft phenotype.
  ○ Records of clinical examinations documenting the presence or absence of associated conditions.

### 2.2 Exclusion criteria

Patients were excluded from the study if any of the following conditions were met:

- Insufficient Clinical Records: Records lacking a definitive OFC diagnosis, clear cleft phenotype description, or documented multidisciplinary assessments were excluded due to the inability to classify the patient's condition and comorbidities accurately.
- Post-Traumatic or Acquired Clefts: Patients with palatal or facial defects resulting from trauma, infection, or tumor resection were excluded.

Initial data collection identified 1,213 potential cases meeting these criteria. However, 26 cases were ultimately excluded due to incomplete data or unclear documentation of OFC status, resulting in a final study population of 1,187 cases.

Data analysis focused on three main areas: determining the prevalence of OFC subtypes from 2011 to 2020, describing the types of associated congenital malformations, syndromes, and medical conditions, and calculating their frequencies and distributions. These calculations primarily involved descriptive statistics to determine proportions, with all frequencies presented as percentages based on the total study population or specific relevant affected subgroups. All statistical analyses were performed using SPSS version 28.0 (IBM Corp., Armonk, NY, USA).

This study was reviewed and approved by the Center for Ethics in Human Research at Khon Kaen University (HE641640). During or after data collection and analysis, authors did not have access to personal information that could identify individual participants with complete anonymization.

## 3 Results

A retrospective review was conducted on 1,187 patients with orofacial clefts (OFCs). The study population consisted of 655 males (55.2%) and 532 females (44.8%). Cleft lip and palate (CLP) was the most prevalent form, accounting for 585 cases (49.2%), followed by cleft palate (CP) with 334 cases (28.1%), and cleft lip (CL) with 267 cases (22.7%).

The majority of cases (*n* = 1,104; 93%) were classified as non-syndromic clefts. Of these, 648 cases (54.6%) were isolated clefts, and 456 cases (38.4%) were associated with additional congenital malformations. Syndromic clefts comprised the remaining 83 cases (7%), further categorized into single gene disorders 36 cases (3.0%) and chromosomal disorders 27 cases (2.3%) (Table 1).

**Table 1:** Characteristics of patients with orofacial clefts at Tawanchai cleft center, Khon Kaen, Thailand, 2011–2020 (*n* = 1,187 cases).

Characteristics	*n* (%)
Gender	
Male	655 (55.2)
Female	532 (44.8)
Age at diagnosis (Days)	
Mean (SD)	183.5 (279.5)
Median (Q1–Q3)	71 (11–204)
Orofacial cleft subtype	
Cleft palate (Q35.1–35.9)	334 (28.1)
Cleft lip (Q36.0–36.9)	267 (22.7)
Cleft lip and palate (Q37.0–37.9)	585 (49.2)
Type	
Non-syndromic (*n* = 1,104, 93.0%)	
Isolated	648 (54.6)
Associated congenital malformations	456 (38.4)
Syndromic (*n* = 83, 7.0%)	
Chromosomal disorders	27 (2.3)
Single gene disorders	36 (3.0)
Others	20 (1.7)
Having medical condition	
Yes	715 (60.2)
No	472 (39.8)

### 3.1 Prevalence and type of associated congenital anomalies

Among 530 children with OFCs and associated congenital anomalies, which included 456 cases with only congenital malformations and 74 cases with syndromes and associated congenital malformations, a total of 813 associated congenital malformations were identified. Respiratory system malformations were the most prevalent (287 cases, 35.3%), followed by circulatory system anomalies (99 cases, 12.2%) and musculoskeletal system anomalies 90 cases (11.1%). When analyzed by cleft type, respiratory system malformations were predominantly occurred in CL and unilateral cleft lip and palate (UCLP) cases, while circulatory and musculoskeletal system anomalies were most frequently observed in CP cases (Table 2).

**Table 2:** Distribution of associated congenital malformations among patients with OFCs according to the affected system, at Tawanchai cleft center, Khon Kaen, Thailand, 2011–2020 (*n* = 530 cases).

Congenital Malformations	Total n (%)	Cleft type				$\chi^{2}$	*p*-value
		CP *n* (%) [95% CI]	CL *n* (%) [95% CI]	BCLP *n* (%) [95% CI]	UCLP *n* (%) [95% CI]		
Respiratory system	287 (35.3)	24 (8.3) [5.6, 12.0]	72 (63.7) [54.5, 72.0]	54 (36.2) [29.0, 44.2]	137 (52.5) [46.4, 58.5]	166.51	<0.001
Circulatory system	99 (12.2)	46 (15.9) [12.1, 20.5]	7 (6.2) [3.0, 12.2]	20 (13.4) [8.9, 19.8]	26 (10.0) [6.9, 14.2]	8.88	0.03
Musculoskeletal system	90 (11.1)	46 (15.9) [12.1, 20.5]	3 (2.7) [0.9, 7.5]	16 (10.7) [6.7, 16.7]	25 (9.6) [6.6, 13.8]	15.50	0.001
Digestive system	63 (7.7)	25 (8.6) [5.9, 12.4]	5 (4.4) [1.9, 9.9]	17 (11.4) [7.2, 17.5]	16 (6.1) [3.8, 9.7]	5.80	0.12
Eye, ear, face, and neck	59 (7.3)	25 (8.6) [5.9, 12.4]	9 (8.0) [4.2, 14.4]	8 (5.4) [2.7, 10.2]	17 (6.5) [4.1, 10.2]	1.89	0.59
Nervous system	43 (5.3)	18 (6.2) [4.0, 9.6]	3 (2.7) [0.9, 7.5]	11 (7.4) [4.2, 12.7]	11 (4.2) [2.4, 7.4]	3.96	0.27
Genital organs	33 (4.1)	10 (3.4) [1.9, 6.2]	5 (4.4) [1.9, 9.9]	7 (4.7) [2.3, 9.4]	11 (4.2) [2.4, 7.4]	0.49	0.92
Urinary system	24 (3.0)	9 (3.1) [1.6, 5.8]	4 (3.5) [1.4, 8.7]	4 (2.7) [1.0, 6.7]	7 (2.7) [1.3, 5.4]	0.26	0.97
Other congenital malformations	115 (14.1)	87 (30.0) [25.0, 35.5]	5 (4.4) [1.9, 9.9]	12 (8.1) [4.7, 13.5]	11 (4.2) [2.4, 7.4]	94.57	<0.001
Total	813 (100.0)	290 (35.7)	113 (13.9)	149 (18.3)	261 (32.1)		

Among 530 cases with congenital malformations that were classified as with and without syndrome, 367 cases (69.2%) presented with malformations in only one system, while 163 cases (30.8%) demonstrated multiple congenital malformations. In cases with multiple congenital malformations, the most common patterns involved two-systems 93 cases (17.5%) and followed by three systems 39 cases (7.4%). Additionally, BCLP and CP cases exhibited the highest prevalence of multiple congenital malformations compared to other cleft types. Notably, two cases exhibited extensive systemic involvement: one right unilateral cleft lip and palate (UCLP) with malformations affecting six organ systems, and one isolated CP case with malformations across seven organ systems (Table 3).

**Table 3:** Distribution of congenital malformations by cleft type in OFC cases, according to the number of affected systems, Tawanchai cleft center, Khon Kaen, Thailand, 2011–2020 (*n* = 530 cases).

Number of congenital malformations	Total n (%)	Cleft type				$\chi^{2}$	*p*-value
		CP *n* (%) [95% CI]	CL *n* (%) [95% CI]	BCLP *n* (%) [95% CI]	UCLP *n* (%) [95% CI]		
1	367 (69.2)	86 (52.1) [44.5, 59.6]	78 (85.7) [77.1, 91.5]	55 (64.7) [54.1, 74.0]	148 (78.3) [71.9, 83.6]	18.51	<0.001
2	93 (17.5)	50 (30.3) [23.8, 37.7]	7 (7.7) [3.8, 15.0]	13 (15.3) [9.2, 24.4]	23 (12.2) [8.2, 17.6]	44.86	<0.001
3	39 (7.4)	19 (11.5) [7.5, 17.3]	4 (4.4) [1.7, 10.8]	6 (7.1) [3.3, 14.6]	10 (5.3) [2.9, 9.5]	11.03	0.01
4	15 (2.8)	5 (3.0) [1.3, 6.9]	1 (1.1) [0.2, 6.0]	5 (5.9) [2.5, 13.0]	4 (2.1) [0.8, 5.3]	4.28	0.24
5	14 (2.6)	4 (2.4) [0.9, 6.1]	1 (1.1) [0.2, 6.0]	6 (7.1) [3.3, 14.6]	3 (1.6) [0.5, 4.6]	7.59	0.09
6	1 (0.2)	0 (0.0) [0.0, 2.3]	0 (0.0) [0.0, 4.1]	0 (0.0) [0.0, 4.3]	1 (0.5) [0.1, 2.9]	1.63	0.59
7	1 (0.2)	1 (0.6) [0.1, 3.4]	0 (0.0) [0.0, 4.1]	0 (0.0) [0.0, 4.3]	0 (0.0) [0.0, 2.0]	2.76	0.45
Total	530 (100.0)	165 (31.1)	91 (17.2)	85 (16.0)	189 (35.7)		

Among the 73 cases with both syndromes and congenital malformations, 21 cases (28.8%) presented with malformations in only one system, while 52 cases (71.2%) demonstrated multiple congenital malformations. In cases with multiple congenital malformations, the most common patterns involved two systems 24 cases (32.9%) and three systems 12 cases (16.4%). Furthermore, CP cases exhibited the highest frequency of syndromic association (35 cases, 47.9%) compared to other cleft types. Notably, six cases (8.2%) exhibited extensive involvement across 5 systems: bilateral cleft lip and palate (BCLP) accounted for 4 cases (23.5%), while CP and UCLP each had 1 case (2.9% and 5.6% of their respective types) (Table 4).

**Table 4:** Distribution of syndromes by cleft type in OFC cases, according to the number of affected systems, Tawanchai cleft center, Khon Kaen, Thailand, 2011–2020 (*n* = 73 syndromic cases with congenital malformations).

Number of congenital malformations	Total n (%)	Cleft type				$\chi^{2}$	*p*-value
		CP *n* (%) [95% CI]	CL *n* (%) [95% CI]	BCLP *n* (%) [95% CI]	UCLP *n* (%) [95% CI]		
1	21 (28.8)	11 (31.4) [18.6, 48.0]	2 (66.7) [20.8, 93.9]	4 (23.5) [9.6, 47.3]	4 (22.2) [9.0, 45.2]	2.83	0.46
2	24 (32.9)	15 (42.9) [28.0, 59.1]	1 (33.3) [6.1, 79.2]	3 (17.6) [6.2, 41.0]	5 (27.8) [12.5, 50.9]	3.58	0.29
3	12 (16.4)	5 (14.3) [6.3, 29.4]	0 (0.0) [0.0, 56.2]	2 (11.8) [3.3, 34.3]	5 (27.8) [12.5, 50.9]	2.66	0.40
4	10 (13.7)	3 (8.6) [3.0, 22.4]	0 (0.0) [0.0, 56.2]	4 (23.5) [9.6, 47.3]	3 (16.7) [5.8, 39.2]	2.78	0.38
5	6 (8.2)	1 (2.9) [0.5, 14.5]	0 (0.0) [0.0, 56.2]	4 (23.5) [9.6, 47.3]	1 (5.6) [1.0, 25.8]	7.05	0.11
Total	73 (100.0)	35 (47.9)	3 (4.1)	17 (23.3)	18 (24.7)		

For specific associated congenital malformations, the three most prevalent associated congenital malformations were nasal deformities (266 cases, 26.0%), Pierre Robin Sequence (PRS: 84 cases, 8.2%), and cardiac septal defects (69 cases, 6.8%). Nasal malformations were most frequently observed in cases of left UCLP, followed by BCLP and right UCLP. PRS, cardiac septal defects [such as atrial septal defect (ASD) and ventricular septal defect (VSD)], along with great artery anomalies [particularly patent ductus arteriosus (PDA)], were predominantly found in CP cases (Table 5).

**Table 5:** Distribution of common associated congenital malformations among orofacial clefts, Tawanchai cleft center, Khon Kaen, Thailand, 2011–2020 (*n* = 530 cases with 1,022 congenital malformations).

Congenital malformation	Total *n* (%)	Cleft type				$\chi^{2}$	*p*-value
		CP *n* (%) [95% CI]	CL *n* (%) [95% CI]	BCLP *n* (%) [95% CI]	UCLP *n* (%) [95% CI]		
Respiratory system							
Nose (Q30)	266 (26.0)	8 (3.0) [1.5, 5.8]	70 (26.3) [21.4, 31.9]	53 (19.9) [15.6, 25.1]	135 (50.8) [44.8, 56.7]	225.86	<0.001
Larynx (Q31)	20 (2.0)	13 (65.0) [43.3, 81.9]	1 (5.0) [0.9, 23.6]	3 (15.0) [5.2, 36.0]	3 (15.0) [5.2, 36.0]	7.83	0.05
Specified congenital malformations affecting multiple systems							
Pierre Robin Sequence (Q87)	84 (8.2)	73 (86.9) [78.1, 92.5]	2 (2.4) [0.7, 8.3]	3 (3.6) [1.2, 10.0]	6 (7.1) [3.3, 14.7]	108.65	<0.001
Circulatory system							
Cardiac septa (Q21)	69 (6.8)	34 (49.3) [37.8, 60.8]	7 (10.1) [5.0, 19.5]	15 (21.7) [13.6, 32.8]	13 (18.8) [11.4, 29.6]	7.38	0.06
Great arteries (Q25)	49 (4.8)	25 (51.0) [37.5, 64.4]	0 (0.0) [0.0, 7.3]	8 (16.3) [8.5, 29.0]	16 (32.7) [21.2, 46.6]	12.52	0.006
Digestive system							
Tongue, mouth and pharynx (Q38)	44 (4.3)	19 (43.2) [29.7, 57.8]	4 (9.1) [3.6, 21.2]	10 (22.7) [12.8, 37.0]	11 (25.0) [14.6, 39.4]	1.96	0.58
Musculoskeletal system							
Feet (Q66)	29 (2.8)	20 (69.0) [50.8, 82.7]	1 (3.4) [0.6, 17.2]	4 (13.8) [5.5, 30.6]	4 (13.8) [5.5, 30.6]	14.97	0.002
Nervous system							
Other brain (Q04)	22 (2.2)	8 (36.4) [19.7, 57.0]	3 (13.6) [4.7, 33.3]	7 (31.8) [16.4, 52.7]	4 (18.2) [7.3, 38.5]	1.75	0.63
Genital organs							
Undescended testicle (Q53)	20 (2.0)	8 (40.0) [21.9, 61.3]	3 (15.0) [5.2, 36.0]	4 (20.0) [8.1, 41.6]	5 (25.0) [11.2, 46.9]	0.22	0.98
Others (Q89)	26 (2.5)	13 (50.0) [32.1, 67.9]	1 (3.8) [0.7, 18.9]	8 (30.8) [16.5, 50.0]	4 (15.4) [6.1, 33.5]	7.11	0.07
Other congenital malformations	393 (38.5)	172 (43.8) [38.9, 48.7]	31 (7.9) [5.6, 11.0]	70 (17.8) [14.3, 21.9]	120 (30.5) [26.2, 35.3]	47.81	<0.001
Total	1,022 (100.0)	393 (38.5)	123 (12.0)	185 (18.1)	321 (31.4)		

Regarding associated medical conditions among children with OFCs, the most common were middle ear infections (614 cases, 51.7%), anemia (205 cases, 17.3%), lack of expected normal physiological development (175 cases, 14.7%), malnutrition (73 cases, 6.1%), and abnormal fetal growth (62 cases, 5.2%). Middle ear infections were notably prevalent; among the types identified, nonsuppurative otitis media accounted for 557 cases and suppurative/unspecified otitis media for 166 cases. Among patients with anemia, the most prevalent types were anemia of chronic disease 46 cases (22.4%), anemia due to enzyme disorders 40 cases (19.5%), and thalassemia 26 cases (12.7%). Short gestation and low birth weight represented a significant proportion of fetal growth abnormalities, occurring in 50 cases (80.6%). Regarding malnutrition, protein-energy malnutrition of mild to moderate degree was the predominant type, presenting in 31 cases (42.5%) (Table 6).

**Table 6:** Distribution of common associated medical conditions in patients with orofacial clefts at Tawanchai cleft center, Khon Kaen, Thailand, 2011–2020 (*n* = 715 cases).

Medical conditions	*n* (%)
Anemia (*n* = 205)	
- Anemia in chronic diseases (D63)	46 (22.4)
- Anemia due to enzyme disorders (D55)	40 (19.5)
- Thalassemia (D56)	26 (12.7)
- Other anemias (D64)	25 (12.2)
- Iron deficiency anemia (D50)	23 (11.2)
- Acute posthemorrhagic anemia (D62)	21 (10.2)
- Anemia, NOS	24 (11.7)
Middle ear infection (*n* = 614) (*152 cases presented both defects)	
- Nonsuppurative otitis media (H65)	557
- Suppurative and unspecified otitis media (H66)	166
Lack of expected normal physiological development (*n* = 175)	175 (100.0)
Abnormal fetal growth (*n* = 62)	
- Short gestation and low birth weight (P07)	50 (80.6)
- Long gestation and high birth weight (P08)	7 (11.3)
- Slow fetal growth and fetal malnutrition (P05)	5 (8.1)
Malnutrition (*n* = 73)	
- Protein-energy malnutrition of moderate and mild degree (E44)	31 (42.5)
- Unspecified severe protein-energy malnutrition (E43)	16 (21.9)
- Unspecified protein-energy malnutrition (E46)	14 (19.2)
- Nutritional marasmus (E41)	8 (11.0)
- Other malnutrition	4 (5.4)

### 3.2 Specific analysis of associated syndromes

Syndromic clefts, identified in 83 patients (7%), were most frequently associated with CP (38 cases, 45.8%), followed by BCLP (21 cases, 25.3%). Among chromosomal disorders, 22q11.2 deletion syndrome was the most common, presenting in 8 cases (9.6%), followed by Down syndrome (6 cases, 7.2%). In the single-gene disorders group, Treacher Collins syndrome was most common (6 cases, 7.2%), followed by Van der Woude and Goldenhar syndromes, each with 4 cases (4.8%). Other notable disorders included VACTERL association (5 cases, 6.0%), and Lennox-Gastaut syndrome (4 cases, 4.8%) (Table 7).

**Table 7:** Distribution of common syndromes associated with orofacial clefts, Tawanchai cleft center, Khon Kaen, Thailand, 2011–2020 (*n* = 83 syndromes).

Syndrome	Total *n* (%) 83 (100)	Cleft type			
		CP *n* (%)	CL *n* (%)	BCLP *n* (%)	UCLP *n* (%)
Chromosomal disorder					
22q11.2 deletion	8 (9.6)	5 (62.5)	1 (12.5)	2 (25.0)	0 (0.0)
Down syndrome	6 (7.2)	2 (33.3)	0 (0.0)	1 (16.7)	3 (50.0)
Other chromosome abnormalities	3 (3.6)	3 (100.0)	0 (0.0)	0 (0.0)	0 (0.0)
Single gene disorder					
Tracher Collins	6 (7.2)	4 (66.7)	0 (0.0)	1 (16.7)	1 (16.7)
Van Der Woude	4 (4.8)	1 (25.0)	1 (25.0)	2 (50.0)	0 (0.0)
Goldenhar	4 (4.8)	0 (0.0)	0 (0.0)	1 (25.0)	3 (75.0)
Frontonasal dysplasia	3 (3.6)	0 (0.0)	0 (0.0)	2 (66.7)	1 (33.3)
Kabuki	3 (3.6)	2 (66.7)	0 (0.0)	0 (0.0)	1 (33.3)
Other					
VACTERL	5 (6.0)	1 (20.0)	0 (0.0)	2 (40.0)	2 (40.0)
Lennox-Gastaut syndrome	4 (4.8)	1 (25.0)	0 (0.0)	2 (50.0)	1 (25.0)
Cornelia De Lange	3 (3.6)	3 (100.0)	0 (0.0)	0 (0.0)	0 (0.0)
Others	34 (40.9)	16 (47.1)	1 (2.9)	8 (23.5)	9 (26.5)
Total	83 (100.0)	38 (45.8)	3 (3.6)	21 (25.3)	21 (25.3)

## 4 Discussion

This 10-year retrospective analysis provides the first comprehensive epidemiological profile of OFCs and their associated anomalies from Northeastern Thailand. Our primary finding reveals a distinct regional prevalence of OFC subtypes, which can now be compared with national and international data. The robustness of this finding is underpinned by the study's design: as the principal tertiary referral center for the region, the Tawanchai Cleft Center captures a large and representative patient cohort, thereby minimizing referral bias. The high case ascertainment observed is a direct result of our integration with a systematic, hospital-wide birth defects surveillance program (15). Critically, the diagnostic validity of the reported anomalies is enhanced by a standardized, multidisciplinary evaluation protocol involving pediatric, obstetric, and clinical genetics specialists (16), lending significant confidence to the observed patterns of comorbidities.

Among the 1,187 patients with OFCs in our study, 54.6% had isolated clefts, representing a lower frequency compared to previous research (70.8%) (17). Additionally, 38.4% of patients with OFCs were found to have associated congenital malformations, which is notably higher than rates reported in several regions, including UAB (33.3%), South Africa (20.0%), Mexico (18.2%), and Britain (16.4%) (7). However, this frequency was still lower than that documented in Jordan (43.3%) (1). Despite the continued lack of comparable epidemiological data on medical conditions in cleft palate within Southeast Asia (18), the high rate observed in our cohort is consistent with findings from other Asian populations, where a similarly high prevalence was reported in the CLP population (19). Furthermore, patients with CLP demonstrated a higher probability of having additional congenital malformations, consistent with findings from both Calzolari and the International Perinatal Database of Typical Oral Clefts (IPDTOC) Working Group (7, 17).

Our finding of a 7% syndromic association rate (83/1,187 cases) in our cohort positions the Northeastern Thai population distinctly within the global epidemiological landscape. This prevalence is remarkably consistent with rates reported in other mixed-ancestry populations, such as Canada (6.2%) and Mexico (6.6%), suggesting a potentially similar etiological architecture. Conversely, our rate is substantially lower than those from predominantly Caucasian cohorts in Britain (13.4%), Australia (11.5%), and Western Europe (10.2%). This striking difference likely reflects a combination of factors, including underlying ethnic and genetic heterogeneity, as well as potential variations in diagnostic protocols and referral patterns across international centers. The significantly lower rates in studies from UAB (0.0%) and Eastern Europe (1.9%) further highlight the profound impact of population-specific and methodological differences on reported outcomes (7).

Among the associated congenital malformations, respiratory system defects emerged as the most prevalent, occurring in 35.3% of cases. This was followed by circulatory system malformations (12.2%) and musculoskeletal system anomalies (11.1%). The strong association with circulatory defects, in particular, is well-documented and reflects the crucial role of cranial neural crest cells, which are not only essential for facial morphogenesis but also for the proper septation of the cardiac outflow tract (20). According to Thai data, the most frequent associated congenital malformations in non-isolated clefts were found in the circulatory system (9.0%), musculoskeletal system (6.3%), and digestive system (5.5%). The first and second most common systems in these findings correspond with our study (9). Studies by Sárközi (21), Hadadi (20), and Yow (22) have consistently shown that congenital heart anomalies were the most common associated condition in patients with OFCs. For instance, a study in another Asian population, which reported high overall comorbidity rates, specifically identified heart defects in 5 out of 132 orofacial cleft patients (19). Additionally, among 575 patients with CLP from Los Angeles, USA, 83 (14.4%) had CHD, and the rates were significantly higher in CLP compared to other cleft types (23), which differs from our study, where CHD rates were significantly higher in patients with CP compared to other cleft types. Another study found that among these patients, the most prevalent malformations were skeletal system abnormalities (27.7%), followed by ocular defects (22.9%), and cardiovascular anomalies (19.3%) (24). The EUROCAT study also reported that musculoskeletal, cardiovascular, and central nervous system defects are frequently associated with CL/P (17), further underlining the critical and frequent association between these conditions across diverse cohorts.

Patients with CLP often develop poor nutrition and anemia due to feeding difficulties. These anatomical abnormalities interfere with nutritive functions, including lactation and mastication, thereby compromising adequate nutrient absorption. When comparing the prevalence of medical conditions between our study and the Burkina Faso study (2007–2014), distinct patterns emerged (1). In our study, middle ear infections were the most common condition (51.7%), with nonsuppurative otitis media. The high prevalence results from anatomical disruptions of the unfused palate and abnormal attachment of the tensor and levator veli palatini muscles, which impairs Eustachian tube function and causes middle ear ventilation dysfunction in cleft patients compared to non-cleft patients (25). Anemia was the second most common condition (17.3%), primarily presenting as anemia of chronic disease. This prevalence was notably lower than the 29.1% reported in an earlier study on children in Thailand (13). Malnutrition was observed in 6.1% of cases, with protein-energy malnutrition of moderate and mild degrees being the most prevalent. In contrast, the Burkina Faso study reported a higher rate of anemia (39.4%) but lower rates of infection (9.2%) and malnutrition (7.5%), along with hemoglobinopathies (4.3%) (1).

Our analysis revealed that Pierre Robin Sequence (PRS) was predominantly associated with CP in 86.9% of cases, while 10.7% presented with CLP. Among these cases, 19 (22.6%) were classified as syndromic PRS. The most frequently observed associated conditions were 22q11.2 deletion syndrome, followed by Treacher Collins syndrome and Stickler syndrome. These findings contrast with studies by Xu (23) and Davies (24), which identified Stickler syndrome as the most prevalent condition, followed by 22q11.2 deletion syndrome.

Syndromic CLP is linked to approximately 400–500 genetic syndromes, with 275 of these having origins in single-gene disorders or chromosomal abnormalities (26). In our study, the most frequently associated syndrome was 22q11.2 deletion syndrome, followed by Treacher Collins syndrome and Down syndrome. This differs from other studies, which PRS and Stickler syndrome were reported as the most common syndromes associated with CP. Additionally, studies by Sárközi (19) and Impellizzeri (26) found Trisomy 13 to be the most prevalent associated syndrome. A separate study at Tawanchai Cleft Center in northeastern Thailand identified syndromic associations in 5 of 123 patients (4%), encompassing Apert syndrome, cleft lip/palate-ectodermal dysplasia, Kabuki syndrome, Oculo-auriculo-vertebral spectrum, and 22q11.2 deletion syndrome (27).

The primary strength of this study lies in its robust sample size and comprehensive case review methodology, where each case underwent meticulous examination and systematic classification. This allowed for a detailed analysis of isolated clefts, associated congenital malformations, syndromic presentations, and concurrent medical conditions. However, we acknowledge several limitations. As a single-center study conducted at a tertiary cleft care facility in northeastern Thailand, the findings may not be fully generalizable to the entire national population. Furthermore, the potential for underdiagnosis of syndromic cases exists, stemming from inconsistent referrals for genetic evaluation and limitations in the availability of advanced chromosomal and gene sequencing analyses. Finally, the retrospective design inherently carries a risk of incomplete data, which may have impacted the reported frequencies of certain conditions.

Despite these limitations, this study provides the first decade-long, hospital-based epidemiological profile of congenital and medical comorbidities in Thai children with OFCs. The inherent retrospective design means we relied solely on documented clinical records, leading to a potential for selection and documentation biases, and notably, resulted in missing genetic confirmation data, especially for a subset of clinically diagnosed non-syndromic cases. The findings carry significant clinical and public health implications. The high prevalence of comorbidities, particularly cardiac defects and middle ear infections, suggests that a multidisciplinary screening protocol should be the standard of care for all newborns with OFCs, rather than an optional addition. Systematically documenting these component anomalies is critical for improving syndrome recognition and delineation, a process essential for accurate diagnosis and genetic counseling (16). The evidence gathered here not only reinforces and validates the comprehensive treatment protocols at the Tawanchai Cleft Center but also provides a foundational dataset to advocate for evidence-based national healthcare strategies, ensuring that all children with clefts in Thailand receive the holistic care they require.

In conclusion, our 10-year analysis confirms that orofacial clefts in the Thai population are not merely structural defects, but represent a complex spectrum of conditions frequently accompanied by significant congenital malformations and medical comorbidities. The high prevalence of associated anomalies, particularly cardiac, otologic, and syndromic presentations, underscores that treating the cleft in isolation is insufficient. This study serves as a compelling call to action for the integration of standardized, multidisciplinary screening and management protocols into the routine care pathway for every child born with an orofacial cleft—a strategy essential for improving long-term health outcomes and ensuring a better quality of life.

## Data Availability

The raw data supporting the conclusions of this article will be made available by the authors, without undue reservation.
